# Outcomes of Endoscopic Third Ventriculostomy in Pediatric Patients With Hydrocephalus

**DOI:** 10.7759/cureus.26608

**Published:** 2022-07-06

**Authors:** Naeem U Haq, Inayat Shah, Muhammad Ishaq, Musawer Khan

**Affiliations:** 1 Department of Neurosurgery, Mardan Medical Complex, Mardan, PAK; 2 Department of Neurosurgery, Bacha Khan Medical College, Mardan, PAK

**Keywords:** ventriculoperitoneal shunt, neural tube defect, endoscopic third ventriculostomy, communicating hydrocephalus, cerebrospinal fluid

## Abstract

Background

Endoscopic third ventriculostomy (ETV) is used to treat patients with obstructive hydrocephalus in infants. This study evaluated the postoperative outcomes of ETV among pediatric patients.

Methodology

A retrospective study was undertaken at the Mardan Medical Complex between June 2018 and June 2021. All pediatric patients who underwent the procedure of ETV in both the absence and presence of choroid plexus cauterization (CPC) at our center were included in the study. Using medical history data, a comprehensive survey questionnaire was designed. The findings and effects were evaluated either as a success or failure.

Results

A total of 90 cases were reviewed during the study. The rate of in-hospital mortality was 1.1% while the most commonly identified causes of hydrocephalus were myelomeningocele and aqueductal stenosis. A total of 39 (43.33%) patients had a successful surgery. In patients where hydrocephalus was secondary to aqueductal stenosis, the success rate was the highest, while the success rate was quite low for post-infectious hydrocephalus and intraventricular hemorrhage (p < 0.0001). The postoperative complication rate was 55.56% in our study. The rate of in-hospital mortality was 1.1%.

Conclusions

We found that the success rate of ETV was dependent upon factors such as the cause of hydrocephalus, type of hydrocephalus, and the age of the patient. Therefore, ETV is not suitable for all patients, and vigilance must be undertaken in selecting patients for the procedure. The rate of postoperative infections in our institution was alarmingly high which is a concerning matter for the institution.

## Introduction

Hydrocephalus is a serious neurological condition affecting infants most frequently [1]. The illness is distinguished by an abnormal build-up of cerebrospinal fluid (CSF), which induces ventricular dilatation and enhanced intracranial pressure, as well as subsequent brain parenchymal atrophy [2]. Two of the standard treatments for hydrocephalus are the placement of a shunt or an endoscopic third ventriculostomy (ETV) [3].

The ventriculoperitoneal shunt (VPS) remains a standard approach toward hydrocephalus due to its safe profile [4]. Other shunting techniques, such as the ventricular atrial shunt and the lateral ventricle-cisterna magna shunt, have been recommended in only selected situations [4]. However, in case of complications, these are very well managed in developed countries. However, the situation is not the same in less developed countries like Pakistan. Bakhsh reported that infants who developed complications due to CSF shunting often presented very late to the healthcare facilities due to socioeconomic conditions and ignorance [5]. The author further highlighted that up to 40% of deaths occurred in infants who had congenital hydrocephalus.

ETV has recently gained popularity among neurosurgeons. It is an alternative procedure in which a bypass is created on the floor of the third ventricle [6]. It is beneficial for patients who have obstructive hydrocephalus. ETV is a treatment of choice in cases of hydrocephalus because it takes away the need for shunt placement [7]. Over the past few years, significant advancements in neuroimaging, endoscopic technology, processing equipment, and a stereotaxic neuronavigation system have resulted in the widespread use of endoscopy for a range of interventions, notably obstructive hydrocephalus, as well as in some selected communicating hydrocephalus [7-9].

Although ETV has already been recognized as an alternative to shunt placement, specifically for young patients with non-communicating hydrocephalus [10], its effectiveness remains questionable, depending on the age and pathophysiology of the hydrocephalus. Therefore, considering the dearth of local literature, this study was conducted to evaluate the surgical outcomes in a cohort of a pediatric population with hydrocephalus.

## Materials and methods

A retrospective study was undertaken at the Mardan Medical Complex between June 2018 and June 2021. After obtaining ethical approval from the institutional review board of Mardan Medical Complex (IRB # NEU-25565), the data acquisition was started.

All records for pediatric patients who underwent ETV in the absence and presence of choroid plexus cauterization (CPC) at our center were included in the study. During the study, 90 cases of hydrocephalus were reviewed who were managed with ETV. Over the period of study, patients were followed up through data obtained from medical records. The documents were evaluated for demographic characteristics, etiology and type of hydrocephalus, overall ETV success rate, associated issues, follow-up, and failure of therapy.

A flexible endoscope telecam and a Bugbee electrocautery wire and a monitor were used for the procedure. The main causes of hydrocephalus were identified. A history of meningitis, ventriculitis, or illness before the development of hydrocephalus; radiographic scans indicating division or partitions in the ventricles; or postoperative observations of hemosiderin or the presence of yellowish deposits were all regarded as post-infectious hydrocephalus. Hydrocephalus secondary to myelomeningocele was defined as the development of the Chiari II malformation. Patients were further subcategorized at the event of ventriculoscopy based on their age and the status of aqueduct.

A conventional ETV via the frontal horn was performed, including fenestration of the third ventricle’s floor. Using electrocautery, short pulses were delivered on the surface. A blunt penetration that fenestrated through Bugbee wire behind the dorsum sellae was performed. By gently stretching the tissues, the wire was employed to eventually dilate the aperture. After ETV, CPC was performed depending on the origin of hydrocephalus. The choroid plexus of the lateral ventricle was completely sealed utilizing the Bugbee wire and a low-voltage monopolar coagulating current, originating at the foramen of monro and subsequently advancing posteriorly.

Followed by procedure, patients were discharged from the hospital after three days and further monitored for one, three, and six months. The patient’s head circumference, fontanel features, signs, neurological assessment, and developmental progress were all evaluated. When considered appropriate, cranial ultrasonography or computed tomography (CT) scans of head were also conducted in certain cases.

The treatment was considered successful if any of the following criteria were met: (i) reduction in growth rate of head circumference growth to normal or less than normal rate, (ii) decompression of the anterior fontanel, (iii) relief from symptoms of elevated intracranial pressure, (iv) resolution of eye findings, and (v) a decrease in ventriculomegaly as identified on neuroimaging. SPSS version 25 (IBM Corp., Armonk, NY, USA) was employed to analyze the retrieved data. For dichotomized variables, a chi-square test was performed. The level of significance was set at 5%.

## Results

A total of 90 cases were reviewed during the study period. Out of these, 11 (12.22%) patients underwent combined ETV and CPC was performed. The majority of the patients were females with a mean age of 12.56 ± 10.12 months. The mean head circumference was found to be 63.44 ± 12.42 cm. Demographic and clinical parameters are illustrated in Table 1. The most common cause of hydrocephalus was myelomeningocele. Other causes included post-infectious hydrocephalus, Dandy-Walker malformation, and space-occupying lesions. The least common cause of hydrocephalus was intraventricular hemorrhage. The most common type of hydrocephalus was non-communicating with a frequency of 58 (64.4%) patients.

**Table 1:** Demographics and clinical parameters of patients with hydrocephalus treated by endoscopic third ventriculostomy.

	N (%)
Age	
Less than 6 months	41 (45.6%)
6–12 months	17 (18.9%)
More than 1 year	32 (35.6%)
Gender	
Female	41 (45.6%)
Male	49 (54.4%)
Causes of hydrocephalus	
Myelomeningocele	34 (37.8%)
Dandy-Walker malformation	8 (8.9%)
Aqueductal stenosis	32 (35.6%)
Space-occupying lesion	5 (5.6%)
Post-infectious hydrocephalus	10 (11.1%)
Intraventricular hemorrhage	1 (1.1%)
Type of hydrocephalus	
Non-communicating	58 (64.4%)
Communicating	19 (21.1%)
Undefined	13 (14.4%)

A total of 39 (43.33%) patients had a successful surgery. In patients where hydrocephalus was secondary to aqueductal stenosis, the success rate was the highest, while the success rate was quite low for post-infectious hydrocephalus and intraventricular hemorrhage (p < 0.0001). Some patients with communicating hydrocephalus were managed with ETV as well. However, the success rate among patients with non-communicating hydrocephalus was significantly greater compared to communicating type (79.49% versus 20.51%; p < 0.0001), as illustrated in Table 2.

**Table 2:** Association of patient outcomes with cause and type of hydrocephalus.

Causes of hydrocephalus	Success (n = 39)	Failure (n = 51)	Total	P-value
Myelomeningocele	9 (23.1%)	25 (49%)	34 (37.7%)	<0.0001
Dandy-Walker malformation	5 (12.8%)	3 (5.9%)	8 (8.9%)	
Aqueductal stenosis	17 (43.6%)	15 (29.4%)	32 (35.6%)	
Space-occupying lesion	5 (12.8%)	0 (0%)	5 (5.6%)	
Post-infectious hydrocephalus	3 (7.7%)	7 (13.7%)	10 (11.1%)	
Intraventricular hemorrhage	0 (0%)	1 (2%)	1 (1.1%)	
Type of hydrocephalus				<0.0001
Non-communicating	31 (79.49%)	25 (49.02%)	56 (62.22%)	
Communicating	8 (20.51%)	26 (50.98%)	34 (37.78%)	

All patients were discharged with a median length of stay of five days. In our study, the postoperative complication rate was 55.56%. Postoperative infection was detected in 70% of cases (35/50). Postoperative bleeding occurred in 13 (26%) cases while two patients had a seizure (Table 3). The rate of in-hospital mortality was 1.1%.

**Table 3:** Postoperative (within six months) complications in patients.

Complication (n = 50)	Incidence
Postoperative infection	35 (70%)
Postoperative bleeding	13 (26%)
Postoperative seizure	2 (4%)

Patients aged fewer than one year who were receiving a combination of ETV and CPC had a considerably better success rate (53%) than patients undergoing ETV alone (25%) in (p = 0.0001) (Table 4). Furthermore, the rate of loss to follow-up at three months was very high at 111 (55.2%).

**Table 4:** Association of age with the success of endoscopic third ventriculostomy.

Patient outcome	Age of patient		Total	P-value
	<1 year (n = 61)	>1 year (n = 29)	90	
Successful	20 (32.8%)	21 (72.4%)	41 (45.6%)	<0.0001
Unsuccessful	61 (67.2%)	8 (27.6%)	49 (54.4%)	

## Discussion

According to the findings, the most commonly reported causes of hydrocephalus were myelomeningocele and aqueductal stenosis, which is in accordance with prior studies [11,12]. Biluts et al. concluded that ETV was performed with satisfactory surgical outcomes and less mortality and morbidity. Similar to our study, Biluts et al. also revealed myelomeningocele and aqueductal stenosis to be the most common causes of hydrocephalus. Furthermore, the authors also found that success rates were significantly higher among one-year-old patients who underwent ETV alone (23%) compared to those undergoing both ETV and CPC (53%) [12]. However, some decades earlier, the literature revealed a different trend in patients with hydrocephalus. For instance, a study by Sacko et al. showed that the most common cause of hydrocephalus between the year 1999 and 2007 was tumors, followed by aqueductal stenosis [13].

In our study, the success of outcomes was significantly lower in patients less than a year old compared to ≥one-year-old patients (p < 0.0001), which is consistent with recent studies [11,12,14]. Madsen et al. reported that ETV was less successful in high-risk causes of hydrocephalus and patients who were younger [14]. Younger patient age and high-risk etiologies were correlated with surgical failure. In contrast, Gangemi et al. revealed that the long-term outcomes of ETV were not affected by the age of the patient as well as the cause of the hydrocephalus [15]. In another study by Brockmeyer et al., the rate of successful ETV varied widely by cause of hydrocephalus and the age of an individual. Patients with aqueductal stenosis and myelomeningocele had the highest success rates. [16]. In our study, postoperative complications included infection, postoperative bleeding, and seizure. The rate of postoperative infections in our institution was alarmingly high which is a concerning matter for the institution. Several others have been reported by Brockmeyer et al. such as transient herniation syndrome, basilar artery perforation, ventriculitis, transient decrease in the level of consciousness, and transient hemiparesis [16]. Strict measures should be taken to avoid postoperative complications in patients who undergo ETV.

In this study, about one-third of patients underwent CPC and ETV, which is the conventional therapy for hydrocephalus among children particularly in underdevelopment nations [17,18]. In most instances, not only will the long-term hazards of shunt reliance be avoided but there also might be numerous advantages. Biluts et al. found that the postoperative infection and surgical causes of death for shunt insertion surgeries were higher in their institution than for the combined ETV and CPC surgery [19]. A thorough meta-analysis was conducted to compare the safety and efficiency of ETV and VPS in patients with hydrocephalus [20]. A total of 546 studies were assessed including four randomized controlled trials. Lu et al. revealed that ETV was associated with a lower occurrence rate of postoperative infection (p = 0.0002), postoperative hematoma (p = 0.03), and blockage rate (p = 0.001) compared with VPS. ETV had no particular impact on death rates (p = 0.06) and occurrence of CSF leakage (p = 0.47) after the surgery in comparison with VPS. However, there were no cases reported of death among patients treated with ETV [20].

On the basis of the current literature and the findings of the study, we recommend that ETV with or without CPC should be performed for pediatric patients with obstructive hydrocephalus as maximum benefits can be obtained employing ETV. However, more studies are needed to ascertain the benefits of ETV in patients with communicating hydrocephalus.

In our study, there were certain limitations due to the retrospective nature of the study. For example, we could not maintain a thorough follow-up of more than six months and were unable to assess the long-term outcomes. Complication management rates in our study could not be assessed because readmission records were not retrievable. Moreover, the one-year mortality rate was not documented as this was a retrospective study, and in our set-up, the records were not maintained properly in many cases.

## Conclusions

In less developed countries, ETV can be undertaken for selected patients with satisfactory outcomes, as reported in our study. However, vigilance must be undertaken, especially in patients who are young as patients aged less than a year were more prone to poorer surgical outcomes compared to older patients. The rate of postoperative infections in our institution was alarmingly high which is a concerning matter for the institution.

## References

[1] Kahle KT, Kulkarni AV, Limbrick DD Jr, Warf BC (2016). Hydrocephalus in children. Lancet.

[2] Krishnan P, Raybaud C, Palasamudram S, Shroff M (2019). Neuroimaging in pediatric hydrocephalus. Indian J Pediatr.

[3] (2022). Hydrocephalus - diagnosis and treatment. https://www.mayoclinic.org/diseases-conditions/hydrocephalus/diagnosis-treatment/drc-20373609#:~:text=The%20most%20common%20treatment%20for,one%20of%20the%20brain's%20ventricles.

[4] Kramer K, Smith M, Souweidane MM (2014). Safety profile of long-term intraventricular access devices in pediatric patients receiving radioimmunotherapy for central nervous system malignancies. Pediatr Blood Cancer.

[5] Bakhsh A (2011). CSF shunt complications in infants--an experience from Pakistan. Pediatr Neurosurg.

[6] Yadav YR, Parihar V, Pande S, Namdev H, Agarwal M (2012). Endoscopic third ventriculostomy. J Neurosci Rural Pract.

[7] Greitz D (2007). Paradigm shift in hydrocephalus research in legacy of Dandy's pioneering work: rationale for third ventriculostomy in communicating hydrocephalus. Childs Nerv Syst.

[8] Boschert JM, Krauss JK (2006). Endoscopic third ventriculostomy in the treatment of shunt-related over-drainage: preliminary experience with a new approach how to render ventricles navigable. Clin Neurol Neurosurg.

[9] (2022). Pacific Adult Hydrocephalus Center. Endoscopic third ventriculostomy. https://www.pacificneuroscienceinstitute.org/hydrocephalus/treatment/endoscopic-techniques/endoscopic-third-ventriculostomy/.

[10] Rasul FT, Marcus HJ, Toma AK, Thorne L, Watkins LD (2013). Is endoscopic third ventriculostomy superior to shunts in patients with non-communicating hydrocephalus? A systematic review and meta-analysis of the evidence. Acta Neurochir (Wien).

[11] Biluts H, Admasu AK (2016). Outcome of endoscopic third ventriculostomy in pediatric patients at Zewditu Memorial Hospital, Ethiopia. World Neurosurg.

[12] Panero I, García-Pérez D, Lagares A (2019). Positive outcome of endoscopic third ventriculostomy in fourth ventricular outlet obstruction. World Neurosurg.

[13] Sacko O, Boetto S, Lauwers-Cances V, Dupuy M, Roux FE (2010). Endoscopic third ventriculostomy: outcome analysis in 368 procedures. J Neurosurg Pediatr.

[14] Madsen PJ, Mallela AN, Hudgins ED, Storm PB, Heuer GG, Stein SC (2018). The effect and evolution of patient selection on outcomes in endoscopic third ventriculostomy for hydrocephalus: a large-scale review of the literature. J Neurol Sci.

[15] Gangemi M, Mascari C, Maiuri F, Godano U, Donati P, Longatti PL (2007). Long-term outcome of endoscopic third ventriculostomy in obstructive hydrocephalus. Minim Invasive Neurosurg.

[16] Brockmeyer D, Abtin K, Carey L, Walker ML (1998). Endoscopic third ventriculostomy: an outcome analysis. Pediatr Neurosurg.

[17] Drake J, Chumas P, Kestle J (2006). Late rapid deterioration after endoscopic third ventriculostomy: additional cases and review of the literature. J Neurosurg.

[18] Oi S, Di Rocco C (2006). Proposal of "evolution theory in cerebrospinal fluid dynamics" and minor pathway hydrocephalus in developing immature brain. Childs Nerv Syst.

[19] Biluts H, Admasu AK (2015). Outcome of ventriculoperitoneal shunt insertion at Myungsung Christian Medical Centre in Ethiopia. East Cent Afr J Surg.

[20] Lu L, Chen H, Weng S, Xu Y (2019). Endoscopic third ventriculostomy versus ventriculoperitoneal shunt in patients with obstructive hydrocephalus: meta-analysis of randomized controlled trials. World Neurosurg.

